# Identifying unstable CNG repeat loci in the human genome: a heuristic approach and implications for neurological disorders

**DOI:** 10.1038/s41439-024-00281-0

**Published:** 2024-06-13

**Authors:** Varun Suroliya, Bharathram Uppili, Manish Kumar, Vineet Jha, Achal K. Srivastava, Mohammed Faruq

**Affiliations:** 1 Department of Neurology, All India Institute of Medical Sciences, Ansari Nagar, Delhi, 110020 India; 2 Genomics and Molecular Medicine, CSIR-Institute of Genomics and Integrative Biology, Mall Road, Delhi, 110007 India; 3 Academy for Scientific and Innovative Research, Ghaziabad, 201002 India; 4 Persistent LABS, Persistent Systems Ltd., Pune, Maharashtra India

**Keywords:** Genomics, Microsatellite instability

## Abstract

Tandem nucleotide repeat (TNR) expansions, particularly the CNG nucleotide configuration, are associated with a variety of neurodegenerative disorders. In this study, we aimed to identify novel unstable CNG repeat loci associated with the neurogenetic disorder spinocerebellar ataxia (SCA). Using a computational approach, 15,069 CNG repeat loci in the coding and noncoding regions of the human genome were identified. Based on the feature selection criteria (repeat length >10 and functional location of repeats), we selected 52 repeats for further analysis and evaluated the repeat length variability in 100 control subjects. A subset of 19 CNG loci observed to be highly variable in control subjects was selected for subsequent analysis in 100 individuals with SCA. The genes with these highly variable repeats also exhibited higher gene expression levels in the brain according to the tissue expression dataset (GTEx). No pathogenic expansion events were identified in patient samples, which is a limitation given the size of the patient group examined; however, these loci contain potential risk alleles for expandability. Recent studies have implicated *GLS, RAI1, GIPC1, MED15, EP400, MEF2A*, and *CNKSR2* in neurological diseases, with *GLS, GIPC1, MED15, RAI1*, and *MEF2A* sharing the same repeat loci reported in this study. This finding validates the approach of evaluating repeat loci in different populations and their possible implications for human pathologies.

## Potential risk alleles in neurological diseases: CNG repeat analysis

Spinocerebellar ataxia and similar muscle and nerve disorders are caused by certain repeated DNA sequences expanding, disrupting normal cell function. In this study, researchers used computer methods to find new DNA repeat expansions that could cause these disorders. They combined computer analysis with genetic testing in a study involving 100 patients with unexplained muscle and nerve disorder symptoms. This method aimed to find cost-effective ways to identify disease-causing genetic changes. The results showed several new unstable DNA repeat expansions that could potentially cause muscle and nerve disorders, although none were definitively linked to the disease in this patient group. The researchers conclude that their method offers a promising way to discover new genetic causes of muscle and nerve disorders. This approach could lead to better diagnosis and understanding of these diseases in the future.

This summary was initially drafted using artificial intelligence, then revised and fact-checked by the author.

## Introduction

Spinocerebellar ataxia (SCA) and other neuromuscular disorders are part of a group of neurodegenerative disorders that share a common disease mechanism of tandem nucleotide repeat expansions^1,2^. The available literature and various databases have identified CNG nucleotide repeats as the most prevalent cause of these neurodegenerative diseases, such as spinocerebellar ataxia^3–5^. Nearly 30-40% of ataxia cases can be explained by trinucleotide CNG repeat expansions^3–5^. Thus, identifying whether CNG expansions in other genes are a causal mechanism for unexplained cases of ataxia and other neuromuscular diseases is imperative.

Earlier gene discovery efforts involving classical gene-mapping efforts identified these CNG expansions as causal events. However, rapidly identifying novel CNG expansions at the cohort level is difficult due to not only the rarity of the disease but also its clinical heterogeneity. Additionally, these genomic regions are considered dark regions, which are inaccessible using traditional methods that utilize short-read sequencing data. The high cost of long-read sequencing, along with time constraints, does not permit a wider scope for identification.

In 2004, Pandey et al. tested a different approach and computationally reviewed the CAG repeats in the entire genome, identifying two CAG loci as putative candidates for SCA^6^.

In this study, we used a combination of computational and genetic methods to identify possible disease-causing unstable repeat loci using a heuristic approach, which may serve as a cost-effective solution.

## Methodology

### Computational approach for TNR screening in the human genome

The FASTA sequences of individual chromosomes in the human reference genome (hg19) were downloaded from the UCSC genome browser (http://hgdownload.soe.ucsc.edu/goldenPath/hg19/chromosomes/). The CNG repeat units associated with various neurological and neuromuscular disorders were selected from the literature for this analysis^1^.

A program to find all possible repeats with a minimum of 4 continuous repetitive units was written in python.

The methodology involved parsing a single whole-genome FASTA file. Using regular expression, repetitive patterns of specified repeat units within each chromosome were identified. Subsequently, a systematic iteration was conducted for each chromosome to detect potential repeat segments, with their repeat complement, including the starting and ending positions from the beginning of the respective chromosome; the length of the repeat; and the repeat unit as the output (source codes are available at https://github.com/bharathramh/STR_repeat/blob/main/str.py). Then, the positions were functionally annotated using the ANNOVAR offline version. We first downloaded the hg19 databases from ANNOVAR, and using the table_annovar.pl command, we annotated the repeat regions.

### Sample enrollment

A total of 100 patients with genetically uncharacterized SCA (retrospective + prospective cases) were enrolled. Patients exhibited autosomal dominant or X-linked inheritance and a sporadic late age of onset and were negative for SCA1, SCA2, SCA3, SCA6, SCA7, SCA8, SCA12, SCA17 and FRDA.

The mean age (SD) of the patients was 59.07 (8.12) (range, 42-84 years), and the mean age at disease onset was 55.54 (7.11) years (range, 42-70 years).

The control samples (N = 100) were obtained from the DNA repository of the Indian Genome Variation Consortium project^7^. We divided the analysis into two stages; the first stage focused on finding the unstable CNG sites in the genome, and the second stage investigated these unstable loci in patients with genetically uncharacterized SCA to identify disease-associated expansion-prone novel repeat loci.

### Evaluation of repeat length variability at the selected loci

A total of 52 loci were targeted for CNG length estimation by polymerase chain reaction (PCR) using an M13-tagged forward primer, a reverse primer, and a fluorescently labeled M13-tagged primer.

For PCR amplification, the sample consisted of template human DNA (25 ng), PCR master mix (Epicentre’s FailSafe mix or Promega master mix), 0.1 µl of forward primer (10 pM/µl), 0.4 µl of reverse primer (10 pM/µl), and 0.4 µl of M13-tagged primer (10 pM/µl) in a reaction volume of 10 µl. The PCR conditions were 95 °C for 3 min; 35 cycles of denaturation at 95 °C for 30 sec, annealing at 60 °C for 30 sec, and extension at 72 °C for 1 min; and a final extension at 72 °C for 5 min. The samples were analyzed using a fragment analyzer and visualized with GeneMapper software (version 4, Applied Biosystems).

## Results

### Identification of CNG repeats from the human reference genome sequence

**Fig. 1:**
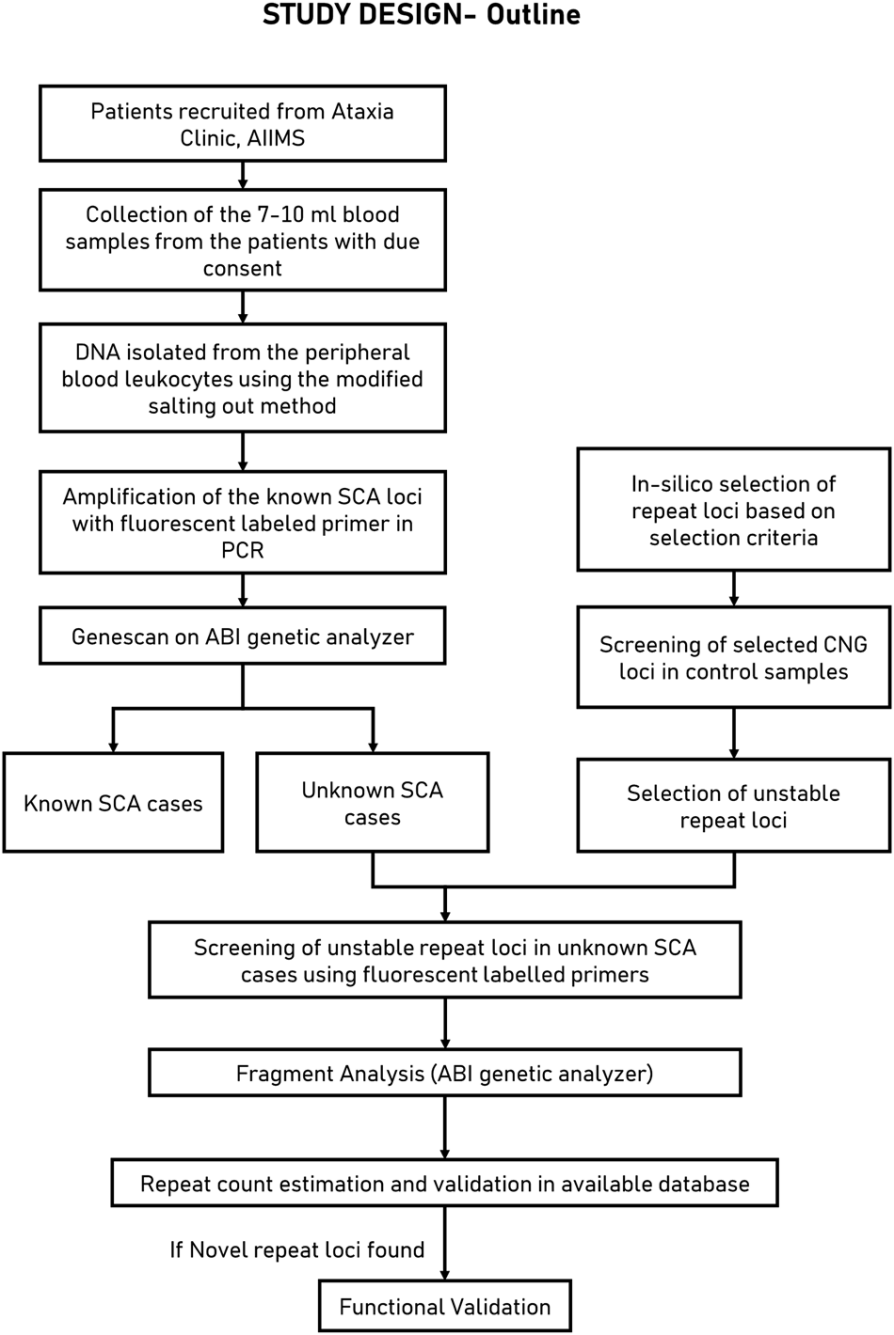
Outline of the study design. In silico selected tandem repeats were assessed for their instability and further screened in patient population of spinocerebellar ataxia disorder.

Through genome-wide CNG repeat selection, we found a total of 15,069 loci ( ≥ 4 contiguous repeats) (Fig. 1). The CNG repeats were abundant in the coding region and UTR. Overall, CAG and CTG repeats were most abundant across different genomic regions (Table 1). We annotated these repeat loci using ANNOVAR^8^ and further categorized the tandem repeats based on the length observed in the reference genome: Group 1, 4–6 repeats; Group 2, 7–9 repeats; and Group 3, >9 repeats (Table 1).

**Table 1:** Categorization of CNG repeat loci based on location and number of repeats in the reference genome.

Repeat Category	Repeat Unit	Coding	Splicing	Exonic; Splicing	3′UTR	5′UTR	5′UTR; 3′UTR	Intergenic	Intronic	ncRNA-Exonic	ncRNA-Intronic	Upstream	Downstream	Upstream/Downstream	Total loci
Group 1	CAG	892	0	0	58	524	1	1566	1355	78	179	267	31	9	4960
	CCG	353	0	1	8	429	0	179	241	29	42	220	2	7	1511
	CGG	410	0	0	11	474	0	187	223	36	20	228	6	7	1602
	CTG	528	5	1	37	61	0	1490	1169	24	156	22	24	1	3518
	GCC	448	0	1	17	491	0	235	299	41	55	283	4	8	1882
Group 2	CAG	82	0	0	3	75	0	86	108	5	11	50	3	0	423
	CCG	28	0	0	1	81	0	17	28	2	3	35	0	1	196
	CGG	34	0	0	0	71	0	14	33	5	0	47	0	0	204
	CTG	53	0	0	3	8	0	70	61	2	12	2	0	0	211
	GCC	34	0	0	1	89	0	20	30	3	3	42	0	1	223
Group 3	CAG	27	0	0	3	14	0	33	25	5	2	11	1	0	121
	CCG	6	0	0	0	16	0	10	3	2	0	17	0	0	54
	CGG	7	0	0	0	11	0	2	10	0	0	10	0	0	40
	CTG	21	0	0	1	2	0	25	13	4	0	1	0	0	67
	GCC	7	0	0	0	18	0	10	3	2	0	17	0	0	57

Group 1 (4–6 repeats), Group 2 (7–9 repeats), Group 3 ( > 9 repeats).

**Fig. 2:**
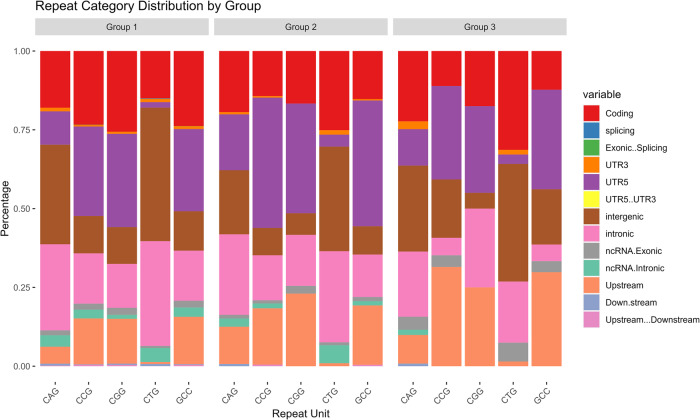
Distribution of repeat categories within groups, showing the percentage of repeats per category, with colors representing different variables.

Using a reductionist approach for further analysis, we selected 52 loci located in CDS region or UTR with a length of contiguous repeats ≥10 (Table 2 and Fig. 2). Repeats with more than 10 units are more prone to expansion events^9^ and cause a decrease in the activity of flap endonuclease-1 (*FEN1*) on Okazaki fragments^10^. Furthermore, most pathogenic trinucleotide repeat expansions were observed in the coding region or UTR, for example, in SCA1-SCA3 (CAG expansion in the coding region), SCA12 (CAG expansion in the 5’ UTR), and myotonic dystrophy (CTG expansion in the 3’ UTR).

**Table 2:** List of 52 selected loci and their repeat status in control samples (unstable loci are marked in bold).

Gene name	Transcript	Chr.	Repeat start	Repeat end	Repeat unit	Region	Reference repeat unit	Repeat variability in controls
*ANKUB1*	NM_001315506	chr3	149484688	149484745	CTG	3’UTR	20	**14–33**
*BACH2*	NM_001170794.1	chr6	90718564	90718564	CTG	5’UTR	10	6–8
*BCL6B*	NM_181844	chr17	6928020	6928047	CAG	CDS	10	15–17
*BMPR2*	NM_001204	chr2	203241237	203241267	CGG	5’UTR	11	15–19
*CAPN6*	NM_014289	chrX	110513720	110513753	CAG	5’UTR	12	11–15
*CASK*	NM_003688	chrX	41376788	41376833	CTG	3’UTR	16	18–23
*CBL*	NM_005188	chr11	119077000	119077034	CGG	5’UTR	12	14–19
*CIZ1*	NM_001131015.1	chr9	130950197	130950197	CTG	5’UTR	10	7–8
*CNKSR2*	NM_014927	chrX	21392714	21392747	CAG	5’UTR	12	**7–17**
*E2F4*	NM_001950	chr16	67229794	67229830	CAG	CDS	13	14–19
*EIF4G3*	NM_001198801	chr1	21502761	21502791	CGG	5’UTR	11	12–18
*EP400*	NM_015409	chr12	132547094	132547133	CAG	CDS	14	**10–21**
*ERF*	NM_001308402	chr19	42758006	42758035	CGG	5’UTR	10	**11–20**
*FAM193B*	NM_001190946	chr5	176981515	176981551	CGG	5’UTR	13	11–17
*FOXP2*	NM_148900	chr7	114055052	114055052	CAG	5’UTR	10	5–6
*GIPC1*	NM_005716	chr19	14606924	14606951	CGG	5’UTR	10	**8–22**
*GLS*	NM_014905	chr2	191745600	191745642	CAG	5’UTR	15	**6–29**
*GSPT1*	NM_002094	chr16	12009243	12009270	CGG	CDS	10	15–18
*HDAC2*	NM_001527.4	chr6	114292073	114292073	CTG	5’UTR	10	8–9
*HTR7P1*	NR_002774.3	chr12	13153376	13153376	CAG	5’UTR	10	**8–17**
*IRF2BPL*	NM_024496	chr14	77492087	77492114	CAG	CDS	10	8–13
*JPH3*	NM_001271604	chr16	87637890	87637933	CTG	CDS	15	**13–24**
*KANSL1L*	NM_001307976	chr2	211035902	211035953	CGG	5’UTR	17	14–18
*LARP6*	NM_197958	chr15	71146430	71146461	CGG	5’UTR	11	12–15
*MAB21L1*	NM_005584	chr13	36050618	36050672	CAG	5’UTR	19	**7–26**
*MAGI1*	NM_001033057	chr3	65425651	65425678	CAG	CDS	10	13–18
*MAML3*	NM_018717	chr4	140812077	140812116	CAG	CDS	14	**18–26**
*MAP3K4*	NM_001291958	chr6	161519351	161519381	CTG	CDS	11	13–16
*MED15*	NM_001293234	chr22	20920814	20920847	CAG	CDS	12	**14–21**
*MEF2A*	NM_001130927	chr15	100252710	100252740	CAG	CDS	11	**13–25**
*MIR205HG*	NM_001104548	chr1	209605639	209605669	CAG	CDS	11	**6–17**
*MLLT3*	NM_001286691	chr9	20413769	20413796	CAG	CDS	10	**12–24**
*MPRIP*	NM_201274	chr17	17039562	17039592	CAG	CDS	11	13–16
*NCOR2*	NM_001206654	chr12	124886962	124886995	CAG	CDS	12	**14–23**
*NPPB*	NM_002521.2	chr1	11918891	11918891	CTG	5’UTR	10	6–8
*PCDH19*	NM_001184880	chrX	99664130	99664157	CGG	5’UTR	10	12–18
*PDCD1*	NM_005018	chr2	242792336	242792367	CTG	3’UTR	11	12–13
*PDS5A*	NM_001100399	chr4	39979441	39979471	CGG	5’UTR	10	13–17
*PIM1*	NM_002648	chr6	37137922	37137922	CAG	5’UTR	10	8–9
*POU6F2*	NM_007252	chr7	39379288	39379315	CAG	CDS	10	13–16
*RAI1*	NM_030665	chr17	17697094	17697130	CAG	CDS	13	**11–17**
*RAPH1*	NM_213589	chr2	204399869	204399905	CAG	5’UTR	10	6–10
*RBM39*	NM_001323423.1	chr20	34328797	34328797	CTG	5’UTR	10	5–7
*RPL14*	NM_001034996	chr3	40503521	40503548	CTG	CDS	10	**13–21**
*S100A16*	NM_001317007	chr1	153579768	153579795	CAG	3’UTR	10	15–16
*TMEM132A*	NM_178031	chr11	60691967	60691998	CGG	5’UTR	10	14–17
*TREML2*	NM_024807	chr6	41159019	41159056	CAG	3’UTR	10	11–12
*UBE2B*	NM_003337	chr5	133707165	133707192	CGG	5’UTR	10	14–17
*UMAD1*	NM_001302350	chr7	7712940	7712998	CAG	5’UTR	20	**15–28**
*USF3*	NM_001009899	chr3	113377918	113377948	CAG	CDS	11	**13–20**
*VEZF1*	NM_007146	chr17	56056545	56056578	CAG	CDS	12	19–21
*ZNF384*	NM_133476	chr12	6777048	6777087	CAG	CDS	14	16–22

### Genotyping of 52 CNG repeats in a control Indian population

**Fig. 3:**
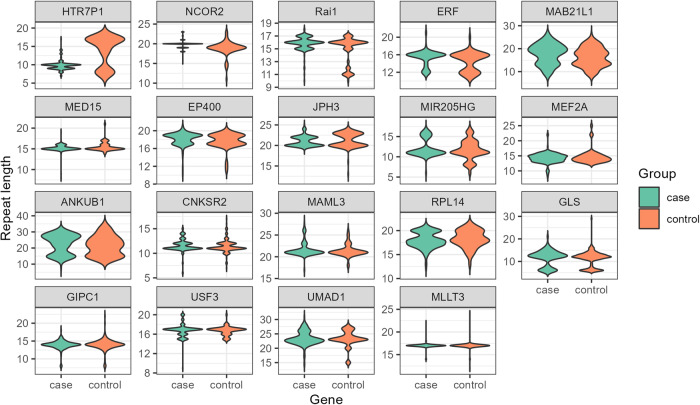
Distribution of target repeats among control and patient samples.

By assessing the length variability of the 52 loci in control samples, 33 loci were found to be relatively stable (length variability of 1–6 repeat units), and 19 loci were more polymorphic in nature (length variability of 7–23 repeat units). These 19 more variable repeat loci (*RAI1, UMAD1, GLS, HTR7P1, CNKSR2, MAML3, MED15, MLLT3, USF3, MEF2A, MIR205HG, NCOR2, RPL14, JPH3, MAB21L1, ANKUB1, ERF, GIPC1*, and *EP400*) were further screened in our ataxia patient cohort to identify any length variation that might be pathogenic (Fig. 3).

The *MAB21L1, ANKUB1*, and *GLS* genes were highly polymorphic and had a wide range of repeat distributions in the population [modes of repeats (ranges): 13 (8–26), 15 (8–33), and 12 (6–29), respectively]. The genes *ANKUB1* and *UMAD1* exhibited a large number of repeats ( > 30 repeats) in both the case and control groups. No significant difference in the large expansion range was observed between the case and control screenings (Table 2).

The heterozygosity indices (HIs, which measure the number of heterozygotes in the population) of *UMAD1, MAB21L1, ANKUB1, GLS*, and *RPL14* were greater than 0.7 in both cases and controls. On the other hand, *MLLT3* and *CNKSR2* were less polymorphic and had more homozygous repeats (HI ≤ 0.1) in both groups. Most of the target loci fell within the range of 0.3 to 0.7, except for ERF, which had an HI of less than 0.25 in all samples.

### Selection of unstable CNG repeats in the 1000 Genomes database

Since disease-associated tandem repeats tend to be more polymorphic in the general population, we investigated the polymorphic nature of these loci in the control population. Compared with different 1000 Genomes control populations, the mode of repeats and variability in the GLS gene were greater in the African and SAS populations (Table 3). *MAB21L1* exhibited a greater repeat range in the EAS population. Although some of the other loci had a maximum of >20 repeat expansions, these loci were uniform or less variable within the populations. *MEF2A* was highly variable, ranging from 2 to 16 repeats, but it was uniform throughout the population. *GIPC1* repeat variability was less common in the EUR population. For *MED15* and *ERF*, repeat data were available for very few patient samples among different populations. We could not find any short tandem repeat data for the *HTR7P1, RPL14, CNKSR2*, or *MLLT3* repeat loci. Our repeat data for the *GLS, ANKUB1, EP400, JPH3*, and *RAI1* loci showed a biallelic distribution, which is also observed in other major populations.

**Table 3:** Features and characteristics of 19 polymorphic loci.

Gene	Group	Reference repeat number	Number of chromosomes	Number of alleles	Range	Mean	SD	Median	Heterozygosity index (HI)	Repeat status in 1000 G	Expression in brain (GTEx)
*ANKUB1*	control	20	52	14	14–33	21.38	5.94	21	0.85	6–26	Present
	case		178	18	8–31	22.49	5.87	24	0.91		
*CNKSR2*	control	12	166	11	7–17	11.42	1.28	11	0.21	–	Present
	case		190	8	6–15	11.57	1.23	11	0.09		
*EP400*	control	14	56	7	11–20	17.71	1.71	17.5	0.54	11–28	Present
	case		188	11	10–21	18.16	1.44	19	0.5		
*ERF*	control	10	128	9	11–20	14.45	2.11	15.5	0.23	6–11	Present
	case		120	8	12–21	15.32	1.71	16	0.25		
*GIPC1*	control	10	168	11	8–22	13.97	1.61	14	0.27	6–22	Present
	case		148	8	8–18	13.90	1.30	14	0.3		
*GLS*	control	15	166	13	6–29	11.28	3.31	12	0.78	5–24	Present
	case		188	12	6–21	11.61	3.40	12	0.89		
*HTR7P1*	control	10	166	6	8–17	13.93	3.93	17	0.61	–	Present
	case		188	9	7–17	9.81	1.30	10	0.52		
*JPH3*	control	15	132	6	13–24	21.30	1.69	20	0.61	5–19	Present
	case		178	7	19–25	21.02	1.20	20	0.56		
*MAB21L1*	control	19	166	18	8–26	16.27	4.07	16.5	0.94	5–26	Present
	case		152	18	8–26	17.70	4.35	19	0.91		
*MAML3*	control	14	58	7	18–26	21.38	1.36	21	0.44	4–20	Present
	case		188	9	17–28	21.65	1.60	21	0.34		
*MED15*	control	12	52	5	14–21	15.50	1.09	15	0.27	9–13	Present
	case		186	7	8–19	15.16	0.87	15	0.33		
*MEF2A*	control	11	52	6	13–25	14.67	2.32	14	0.69	2–14	Present
	case		170	10	8–22	14.27	1.70	14	0.61		
*MIR205HG*	control	11	54	10	6–17	11.67	2.37	11	0.44	3–17	Not Present
	case		184	7	6–17	12.19	2.15	11	0.46		
*MLLT3*	control	10	180	7	12–24	16.97	0.77	17	0.07	–	Present
	case		190	5	14–22	16.97	0.59	17	0.05		
*NCOR2*	control	12	158	9	11–23	18.86	1.79	19	0.46	4–15	Present
	case		176	7	15–23	19.88	1.04	20	0.51		
*RAI1*	control	13	90	7	10–17	14.87	2.09	16	0.34	6–14	Present
	case		184	9	10–18	15.86	1.03	16	0.46		
*RPL14*	control	10	52	8	13–21	18.25	1.78	18	0.69	–	Present
	case		190	10	12–21	17.99	1.65	18	0.79		
*UMAD1*	control	20	52	9	15–28	23.31	2.84	23	0.62	4–24	Present
	case		190	16	14–31	23.41	2.39	23	0.78		
*USF3*	control	11	66	5	15–20	16.71	0.89	17	0.52	6–16	Present
	case		184	7	9–20	16.78	1.30	17	0.62		

Interestingly, we observed variability in the repeat ranges of *USF3, MEF2A, JPH3, RAI1, ERF, MED15, MAML3*, and *UMAD1* compared to those of other world populations, but none of the differences were significant according to the Wilcoxon signed rank test (nonparametric test). Both our groups had comparatively fewer repeats for EP400 loci (Table 4). The probable reason for this difference is the use of different sequencing technologies; short-read sequencing was employed for the 1000 Genomes Project data. While short-read sequencing has its advantages, it also has some inherent inefficiency in regard to capturing long-range repeats and complex genomic regions.

**Table 4:** Repeat length variability in 1000 Genome subpopulations.

S No.	GENE	AFR	AMR	EAS	EUR	SAS	Control	Case
1	*MIR205HG*	13 (4–18)	9 (4–18)	9 (3–14)	9 (3–14)	9 (4–16)	11 (6–17)	11 (6–17)
2	*GLS*	14 (5–24)	8 (5–23)	8 (6–21)	8 (6–22)	14 (6–26)	12 (6–29)	12 (6–21)
3	*USF3*	13 (6–15)	13 (7–17)	13 (8–16)	13 (6–16)	13 (6–17)	17 (15–20)	17 (9–20)
4	*ANKUB1*	11 (8–25)	11 (9–27)	11 (6–26)	11 (7–26)	11 (6–26)	15 (14–33)	15 (8–31)
5	*MAML3*	18 (4–20)	18 (8–19)	18 (4–18)	18 (6–19)	18 (8–18)	21 (18–26)	21 (17–28)
6	*UMAD1*	18 (7–25)	17 (6–25)	17 (4–24)	17 (6–24)	17 (6–25)	23 (15–28)	23 (14–31)
7	*NCOR2*	17 (10–21)	18 (13–22)	18 (10–21)	18 (4–22)	18 (11–22)	19 (11–23)	20 (15–23)
8	*EP400*	28 (12–29)	28 (12–29)	28 (12–29)	28 (11–29)	28 (12–29)	17 (11–20)	19 (10–21)
9	*MAB21L1*	11 (5–27)	13 (6–26)	17 (4–26)	13 (6–27)	13 (5–26)	13 (8–26)	13 (8–26)
10	*MEF2A*	11 (2–16)	11 (2–13)	9 (2–16)	11 (3–13)	9 (2–14)	14 (13–25)	15 (8–22)
11	*JPH3*	15 (6–26)	15 (9–19)	15 (10–19)	15 (10–20)	15 (7–25)	20 (13–24)	20 (19–25)
12	*RAI1*	12 (6–15)	12 (7–15)	12 (5–13)	12 (6–14)	12 (6–14)	16 (10–17)	16 (10–18)
13	*GIPC1*	13 (6–22)	14 (9–17)	14 (6–18)	14 (12–20)	14 (7–22)	14 (8–29)	14 (8–18)
14	*ERF*	9 (6–10)	9 (8–10)	9 (9–9)	9 (8–11)	9 (6–11)	16 (11–20)	16 (12–21)
15	*MED15*	12 (12–12)	12 (12–12)	12 (12–12)	12 (9–13)	12 (11–12)	15 (14–21)	15 (8–19)

### Analysis of the expression levels of genes harboring unstable repeats

For all the candidate genes, the bulk tissue gene expression of each gene was compared among different tissues using GTEx^11^. The analysis showed that the *CNKSR2, MAB21L1, USF3, RAI1, NCOR2, JPH3, MAML3, EP400*, and *GLS* genes were significantly highly expressed in the brain, particularly in the cerebellum. All the other genes, except for *MIR205HG*, also exhibited significant expression levels in the brain (Table 2). Since the pathogenesis of SCA is associated with the brain, we excluded *MIR205HG* from the gene shortlist. Thus, we proposed the pathogenicity of the remaining 18 genes, which might show an ataxia phenotype.

## Discussion

Repeat instability is an underlying mutation mechanism for several neurodegenerative disorders in humans. Understanding the mechanism of repeat instability in disease manifestation has always been challenging. Several distinct hypotheses on repeat expansion have been proposed over the years, but its mechanism is not fully understood^2,12–15^. Repeat instability in spinocerebellar ataxia is the most prevalent genetic manifestation worldwide. Identifying repeat expansion regions has always been challenging. In recent years, long-read next-generation sequencing has been an effective method for identifying these targets, but this method is costly and requires a large setup and personnel with highly qualified expertise. Here, we used a cost-effective alternative approach for the investigation of tandem nucleotide repeats.

The initial phase of the study utilized a computational approach, yielding 52 suspected CNG repeat loci from various genes for further investigation in the Indian control population. Using a cost-effective fluorescent PCR-based fragment analysis approach identified 19 conclusive highly polymorphic repeat targets after screening the control samples.

Genetic markers for the same disorder have been shown to be expressed among various populations in diverse ways, with some diseases and genetic markers being population specific. Therefore, in the second phase of the study, we screened these putative candidates in patients with genetically uncharacterized clinically confirmed SCA. Although no large expansion of these target loci was identified in the study population, repeat polymorphisms in other populations of the 1000 Genomes Project were used as a proof of concept. We evaluated all identified unstable markers in different major populations and our control and patient samples to understand the population variability among these loci. We found repeat data for 15 of the 19 selected CNG loci in the 1000 Genome STR database.

Additionally, the GTEx data showed that, except for *MIR205HG*, the remaining 18 loci were expressed in various brain tissues, making them more suitable for further investigations. However, of the 18 identified highly unstable repeat loci, none exhibited large repeat expansions in our patient population.

Multiple studies published in recent years on point and repeat expansion mutations for various neuro-related disorders from the proposed list of 18 genes support our adopted strategy in this study^16–19^. Variation in the length of CAG repeats in the *RAI* gene is associated with differences in age at onset in spinocerebellar ataxia type 1 patients among various populations^16^. In 2019, Rad et al. reported that a point mutation in *MAB21L1* causes a syndromic neurodevelopmental disorder with distinctive cerebellar, ocular, craniofacial, and genital features (COFG syndrome)^20^. Another study suggested that a point mutation in *CNKSR2* is associated with seizures and mild intellectual disability^20^. In 2020, a report was published suggesting that frameshift mutations of *GLI3, ANKUB1*, and *TAS2R3* might alter protein functions and accelerate the progression of polysyndactyly (PSD), an autosomal dominant genetic limb malformation^21^. The *EP400* gene has been proposed to play a significant role in oligodendrocyte survival and myelination in the vertebrate central nervous system^22^. One study proposed that differences in the polyglutamine repeat length in *MED15* change the expression of diverse stress pathways^17^. In various populations, CAG repeat variation in *MEF2A* is a risk factor for coronary artery disease (CAD)^18^. A study published in 2020 suggested that a CGG repeat expansion mutation in the 5’UTR of *GIPC1* causes oculopharyngodistal myopathy (OPDM), an adult-onset inherited neuromuscular disorder^18^. A large GCA tandem expansion in the 5’ UTR of the *GLS* gene causes overall developmental delay, progressive ataxia, and elevated levels of glutamine^19^. Reported studies of *GLS, GIPC1, MED15, RAI1*, and *MEF2A* included the same candidate loci that we identified in our study^7,13–15^. Although we did not identify any large repeat expansions, this previously reported evidence strengthens our study, indicating that our approach is in the right direction for the discovery of novel targets.

### Limitations of the study

1. We considered only CNG repeats in the coding region and UTR with at least 10 continuous repeats due to the larger number of target loci. Considering other tri-, tetra-, penta-, and hexa-repeat units and loci with lower repeat numbers increases the chances of obtaining causal mutations.
2. We collected 100 patient samples for the study. SCA is a rare disorder, and its subtypes are very rare; therefore, a larger sample size will provide more confidence in our hypothesis.
3. Most SCA subtypes are geographic and population specific. In this study, we considered only North Indian SCA patient samples, and a multipopulation study could enhance the possibility of identifying causal mutations among the studied genes.

This study highlights the importance of the population polymorphism approach for understanding the genetic background and mechanism of tandem repeat instability in ataxia-like neurological disorders. The role of other repetitive sequences in both coding and noncoding regions in the context of neurological disorders can be explored with the help of computational and polymorphism approaches, as in this work.

## Conclusion

Although our study did not positively identify any novel pathogenic CNG trinucleotide repeat expansions, it still describes an approach that utilizes population-level genomics data to address the complex genetic mechanisms underlying disease pathology. The list of novel unstable loci that we identified can be examined in other neurological and neuromuscular disease cohorts, and a larger sample size may lead to the discovery of pathogenic expansions at these loci.

## Supplementary information

Supplementary Files

## Data Availability

The data from this study related to the subjects and code will be available upon request to the corresponding author. The codes utilized in the study are available at https://github.com/bharathramh/STR_repeat/blob/main/str.py).
